# Adsorption of citrate ions on hydroxyapatite synthetized by various methods

**DOI:** 10.1007/s10967-013-2825-z

**Published:** 2013-11-22

**Authors:** E. Skwarek, W. Janusz, D. Sternik

**Affiliations:** Faculty of Chemistry, Maria Curie-Sklodowska University, Maria Curie-Sklodowska Sq. 5, 20-031 Lublin, Poland

**Keywords:** Hydroxyapatite, Adsorption, Citric acid, Thermal analysis

## Abstract

The specific adsorption of citric acid ions at hydroxyapatite interface was investigated by the means of radioisotope method (^14^C) as a function of citric acid ions concentration, NaCl concentration and pH. Application of the hydroxyapatite has become wide in the biomaterial field as the Ca_10_(OH)_2_(PO_4_)_6_ possess biocompatibility with human hard tissue. Hydroxyapatite was synthesized using three different methods. The physical properties of the resulting powder were characterized by DTA/TG, XRD, AFM and SEM microscopy. Physicochemical qualities characterizing the electrical double layer of the hydroxyapatite/NaCl solution interface were determined. The zeta potential and the adsorption of citric acid molecule were studied as a function of pH. The point of zero charge and the isoelectric point of samples were determined. Electrical double layer parameters of hydroxyapatite/NaCl interface are influenced by a synthesis method. The points pH_pzc_ and pH_IEP_ for sample 1 are pH_pzc_ 7.5 and pH_IEP_ 3; for sample 2 pH_pzc_ 7.05 and pH_IEP_ 3, for smaple 3 pH_pzc_ 6.7 and pH_IEP_ 3. Temperature has weak influence both on pure substance and with citric acid adsorbed, as derivatographic analysis has shown, and characterization of hydroxyapatite structure may be carried out by this thermal analysis. Two phenomena are responsible for citric acid adsorption: phosphate group’s replacement at hydroxyapatite surface by citric ions parallel to intraspherical complexes formation.

## Introduction

Hydroxyapatite [Ca_10_(OH)_2_(PO_4_)_6_] has been studied for many years, due to its occurrence in natural environment and practical application. As a natural mineral it is present in igneous and metamorphic rocks. Moreover, it is a major inorganic component of bones and teeth. Calcium phosphate and calcium carbonate are human hard tissues constituents, with the same structure as the natural mineral. Thanks to its crystallographic similarity with natural bone minerals, biocompatibility with hard tissues and with skin or muscle tissues, bioactivity, no toxicity, and implants made of synthetic hydroxyapatite (bioceramic hydroxyapatite) are used as an artificial bone substitute in orthopedic, neurosurgery, dental surgery, plastic surgery and dental applications [1–4].

Methods of hydroxyapatite preparation may have influence on specific chemical properties of adsorbent surface. High purity and desirable microstructure of hydroxyapatite have been obtained using the processes: sol–gel, co-precipitation, microemulsion synthesis or hydrothermal reactions [5]. The study of the electrical double layer structure for the hydroxyapatite/electrolyte solution interface reveal that the pH_pzc_ and pH_iep_ data lie in a wide range, from 4.35 for dental hydroxyapatite up to 8.6 for synthetic samples, and it is very difficult to compare them. The main reason for this is the fact that all experimental results were obtained for hydroxyapatite samples of different origin, stoichiometry and purity, under various experimental conditions, and by different experimental techniques. However, the solubility of hydroxyapatites should be taken into account in determination of surface charge density and pH_pzc_ by means of potentiometer titration. The studies of the electrical double layer at the hydroxyapatite/electrolyte solution interface show that besides Ca^2+^ and phosphate, H^+^ and OH^−^ are potential determining ions [6, 7]. The charge on hydroxyapatite arises as a result of various reactions of surface hydroxyl groups and unequal adsorption from solution of ions of opposite charges [8–10]. Because hydroxyapatite is a basic salt, there are two types of groups on the surface: hydroxyl and phosphatic. There is no doubt that the phosphatic groups have more acidic character than hydroxyl ones, which are of alkaline character. The reactions of surface groups at the hydroxyapatite that create surface charge are following:

1 $$ \equiv {\text{CaOH}} + {\text{H}}^{ + } \rightleftarrows \equiv {\text{CaOH}}_{2}^{ + } $$

2 $$ \equiv {\text{CaOH}} \rightleftarrows \equiv {\text{CaO}}^{ - } + {\text{H}}^{ + } $$

3 $$ \equiv {\text{PO}}_{4} {\text{H}} \rightleftarrows \equiv {\text{PO}}_{4}^{ - } + {\text{H}}^{ + } $$

4 $$ \equiv {\text{PO}}_{4}^{ - } + {\text{Ca}}^{2 + } \rightleftarrows \equiv {\text{PO}}_{4} + {\text{Ca}}^{2 + }. $$

The literature about sorption on hydroxyapatite using the isotope method includes the papers about adsorption of Ni, Cu, and Sr written by Rosskopfova et al. [11–13]. However of no less importance are problems related with anions, among others, citrate anions.

The adsorption process of citric acid on the surface of hydroxyapatite was described by Filgueiras, Mkhonto and de Leeuw using the computer modelling method. The structure and energy of the hydroxyapatite and citric acid complex was determined thanks to interatomic potentials. There was a series of computer simulations performed to investigate the adsorption of citric acid on different surfaces of hydroxyapatite. They showed that the intensity of the acid particle influence on the surface is conditioned by stability of the surface and possibility of repeated interaction of an acid molecule with the active adsorption places of hydroxyapatite, especially with two or more calcium atoms. Big adsorption energy causes the citric acid adsorption on the surface of hydroxyapatite to be exceptionally strong. Therefore it can be concluded that citric acid should serve as a good inhibitor for the hydroxyapatite increase if we look at it as to be used in the synthesis. Blocking the active places on the surface of hydroxyapatite inhibits the increase of its crystal [14].

While investigating the citric acid adsorption on hydroxyapatite, Vega, Narda and Ferreti [15] explained the process in a following way. The adsorption process occurred through the exchanges of phosphate groups with citrate anions. The adsorption isotherm equalled the Langmuir isotherm. The complex of citric acid and hydroxyapatite originated from combining one molecule of citric acid with two active places of hydroxyapatite. It was confirmed during research that the active places on the surface of hydroxyapatite are not equal and the surface itself is not uniform. The special structure of hydroxyapatite caused the fact that, the possibility of the ions adsorption depends on the occupied neighbouring spaces. In connection to that the adsorption of the first layer of citrate at the hydroxyapatite/nonelectrolyte solution interface can be treated as monolayer adsorption and the next layers become a part of the solution itself. As Lopez-Macipe, Gomez-Moralez and Rodriguez-Clemente [16] explain in their work, the adsorption of citric acid on hydroxyapatite depends to a great extent on pH value. However, the mechanism of interactions between citric acid and hydroxyapatite is not completely clear. There is a theory which states that citric acid ions influence hydroxyapatite through ionic exchange between phosphate groups and citrate ions. In reactions between citrate ions and hydroxyapatite, pH value has a crucial value because it determines the kinds of citric ions capable of adsorption on the hydroxyapatite surface. According to the authors’ assumptions citrate ions can have different forms e.g. Cit^3−^, HCit^2−^ and H_2_Cit^−^. The dependence was determined on the basis of the analysis concerning the citrate ions on hydroxyapatite at of 25 and 37 °C. Amount of citric ions adsorbed was different in examined solutions because of different pH values. At pH value 6 the dominating form were H_2_Cit^-^ ions whereas at pH value 8 there was the highest concentration of Cit^3−^ ions. The adsorption process of citric acid on hydroxyapatite with low concentration of citric acid was described using the Langmuir adsorption model. This adsorption occurs here through replacement of the phosphate ions by citrate ions on the solid substance/solution interface. This is a result of higher relation of citrate ions to the hydroxyapatite surface. Presence of different forms of citrate ions caused different types of interactions. Cit^3−^ ions react in a two-donor way (1 citrate ion occupies 2 sites of Ca) and reactions of HCit^2−^ ions are stronger than the previous one (1 citrate ion replaces 1 Ca). Such dependence is possible owing to the resonance between the monodonor (using one group –COO^−^) and the surface of chelation (using two groups –COO^−^).To sum up, the adsorption decreases with the growth of pH value. Despite that the experiments showed that at pH 6 it is higher than in a solution at pH 8 [17]. Chemical reactions between citric acid and hydroxyapatite in a human body have many consequences. The first one is partial dissolution of apatite, another one is precipitation of calcium citrate occurring when the concentration of the acid is over 10 mmol/dm^3.^ According to Misra, interactions between citric acid and hydroxyapatite can be called chemical reactions. However, the exchange with phosphate ions is responsible for the adsorption of citrate ions. This is confirmed by short time of the balancing process during the reaction of citrate salts of alcaline metals with hydroxyapatite. The literature has largely demonstrated that the synthesis routes and related parameters could significantly affect not only hydroxyapatite particle size and morphology, but also phase composition, thermal stability as well as sintering behavior [18].

The information available in the literature concerning hydroxyapatite focuses mainly on two issues: thermal analysis of hydroxyapatite and electrochemical properties analysis. This work is combination of these two scientific currents using the example of hydroxyapatites obtained in three different syntheses, which makes a wider view possible on hydroxyapatite as biomaterial used by the human body.The goal of this paper is characterization of hydroxyapatite, determination of pH_pzc_ and pH_iep_ points, establishing zeta potential depending on pH. The specific adsorption of citric acid ions at the hydroxyapatite interface was investigated. The main part of the study was to investigate the thermal effects: caused by citric acid sorption on hydroxyapatite and methods of hydroxyapatite preparation.

## Materials and method

The investigated adsorbents were synthesized by three wet methods [2–4].

In this paper hydroxyapatite was prepared using three methods:

### Method 1 [2]

5 $$ 10{\text{ Ca}}\left( {\text{OH}} \right)_{ 2} + {\text{ 6 H}}_{ 3} {\text{PO}}_{ 4}   \to {\text{Ca}}_{ 10} \left( {{\text{PO}}_{ 4} } \right)_{ 6} \left( {\text{OH}} \right)_{ 2} + 1 8 {\text{ H}}_{ 2} {\text{O}} $$

In the reaction there were used the following reagents: Calcium hydroxide Ca(OH)_2_ produced by Aldrich and phosphoric acid H_3_PO_4_ (V) made by POCh Gliwice. There were prepared 1 M aqueous solutions with reagents, 0.3 dm^3^of phosphoric acid and 0.18 dm^3^ of calcium base were used in the reaction. The H_3_PO_4_ solution was dropped into the Ca(OH)_2_ suspension placed in the flask for 15 min. During dropping in the reaction mixture was stirred vigorously and then dried in a dryer for 24 h. A white sediment of the crystalline structure of hydroxyapatite was obtained. Then the sediment was washed with redistilled water till the constant value of redistilled water conductivity was achieved.

### Method 2 [3]

In the reaction there were used the following reagents: calcium acetate (CH_3_COO)_2_Ca from Fluka and dipotassium hydrophosphate K_2_HPO_4_ from POCh, Gliwice. The solutions of concentrations 0.1 M K_2_HPO_4_ and 0.06 M –(CH_3_COO)_2_Ca were prepared. In the reaction there was taken 0.15 dm^3^ of each salt and both solutions were dropped into 0.2 dm^3^ of water placed in the reaction flask. The flask was immersed in a water bath heated up to 100 °C. The salt solutions were dropped in at the same time for 30 min and then the reaction mixture was boiled for 1 h. The mixture was stirred vigorously and the constant temperature was kept all the time. The obtained sediment was washed with redistilled water till the constant value of redistilled water conductivity was achieved.

### Method 3 [4]

6 $$ 6{\text{CaHPO}}_{4} \cdot 2{\text{H}}_{2} {\text{O}} + 4{\text{Ca}}({\text{OH}})_{2} \to {\text{Ca}}_{10} ({\text{PO}}_{4} )_{6} ({\text{OH}})_{2} + 18{\text{H}}_{2} {\text{O}}. $$

### Synthetic hydroxyapatite was prepared hydrothermally according to the reaction

In order to prepare hydroxyapatite there was applied the following procedure: 1.0324 g of CaHPO_4_ *2 H_2_O from Fluka and 0.2964 g of Ca(OH)_2_ from Aldrich were taken and mixed. 80 ml of double distilled water was poured in and the pH value was reduced to 9 using 80 % analytically pure acetic acid from POCh. The reaction mixture was put into a drier for 24 h at 120 °C. The obtained hydroxyapatite had to be purified from reagents so the sediment was washed many times with redistilled water and centrifuged.

Hydroxyapatite was investigated using X-ray diffraction (XRD), adsorption–desorption of nitrogen accelerated surface area and porosimetry (ASAP2405, Micromeritics Instruments, Co.), photon correlation spectroscopy (PCS), AFM and SEM microscopes. The classical thermogravimetric (aparatQ-1500D type made by MOM, Budapest, Hungary) TG and DTG curves of thermal analysis, which gave the dependence of the weight loss of a sample as a function of temperature or time, were measured over a temperature range from 20 to 1,000 °C with a furnace-heating rate of 10 °C/min. The TG and DTG curves were registered digitally under the control of the program Derivat running on PC.

The specific adsorption of citric acid ions at the hydroxyapatite interface was investigated by the means of the radioisotope method as a function of citric acid ions concentration, NaCl and pH. The initial concentration of citric acid ions ranged from 1 × 10^−6^ to 1 × 10^−3^ mol dm^−3^, pH was changed from 6 to 12. As a background electrolyte NaCl solution was used of the concentrations 0.001 mol dm^−3^. The adsorption measurements were complemented by the potentiometric titration of hydroxyapatite suspensions and electrophoresis measurements.

To remove ionic type contaminations, which might influence the ion adsorption measurements, the hydroxyapatite was washed with double distilled water until constant conductivity about 0.5 μS/cm was achieved. Adsorption and surface charge measurements were performed simultaneously in the suspension of the same solid content, to keep the identical conditions of the experiments in a thermostated Teflon vessel at 25 °C. To eliminate the influence of CO_2_ all potentiometric measurements were performed under nitrogen atmosphere. pH values were measured using a set of glass REF 451 and calomel pHG201-8 electrodes with the Radiometer assembly. Surface charge density was calculated from the difference of the amounts of added acid or base to obtain the same pH value of suspension as for the background electrolyte.

The zeta potential of hydroxyapatite dispersions was determined by electrophoresis with Zetasizer 3000 by Malvern. The measurements were performed at 100 ppm solid concentration ultrasonication of the suspension.

## Results and discussion

The specific surface of the hydroxyapatite samples was obtained by adsorption–desorption of nitrogen. Table 1 presents the structural parameters of hydroxyapatite. On the basis of the results in Table 1, the difference in the surface area and average volume of pores can be seen. The surface area and average volume of pores of sample 1 are greater than those of samples 2 and 3. The analyzed samples of hydroxyapatite have mesopores, a significant difference in the size of the surface itself, which confirms the influence of the synthesis method, can be also observed.

**Table 1:** Chosen structural parameters, sample 1, sample 2 and sample 3

	Sample 1	Sample 2	Sample 3
BET surface area (m2/g)	86.15	34.42	26.34
Langmuir surface area (m2/g)	109.91	44.25	33.71
BJH cumulative adsorption surface area of pores between 1.7 and 300 nm diameter (cm3/g)	0.51	0.14	0.13
BJH cumulative desorption surface area of pores between 1.7 and 300 nm diameter (cm3/g)	0.50	0.13	0.12
Average pore diameter (4 V/A by BET) (nm)	18.81	12.24	15.08
BJH adsorption on average pore diameter (4 V/A) (nm)	20.82	13.55	15.75
BJH desorption on average pore diameter (4 V/A) (nm)	19.08	13.48	8.39

**Fig. 1:**
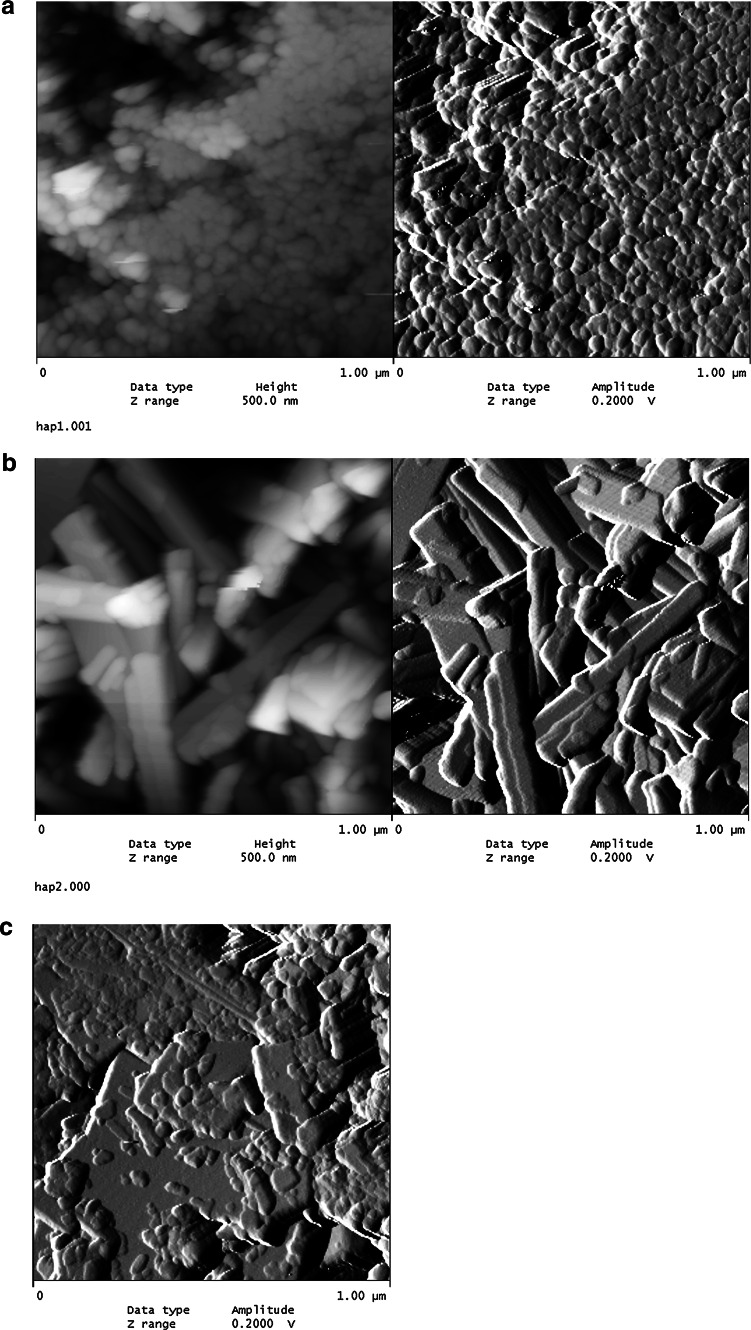
AFM micrograph of hydroxyapatite samples 1 (**a**), 2(**b**), 3(**c**)

Figure 1 shows the AFM micrographs of several hydroxyapatite samples 1, 2, and 3. The examination with an AFM microscope confirms the porosity of the synthesized hydroxyapatites samples of surface. At the pictures you can see the structures built from little crystals with visible pores. Topographic analysis of the hydroxyapatite samples shows that the surface of sample 2 is rougher, the average roughness index is 44 whereas for sample 1 it is 30.4 and for sample 3–21.28. The average roughness for sample 2 is 172 nm, for sample 1-149 nm and for sample 3–142 nm. Sample 2 exhibited the formation of whisker morphologies.

**Fig. 2:**
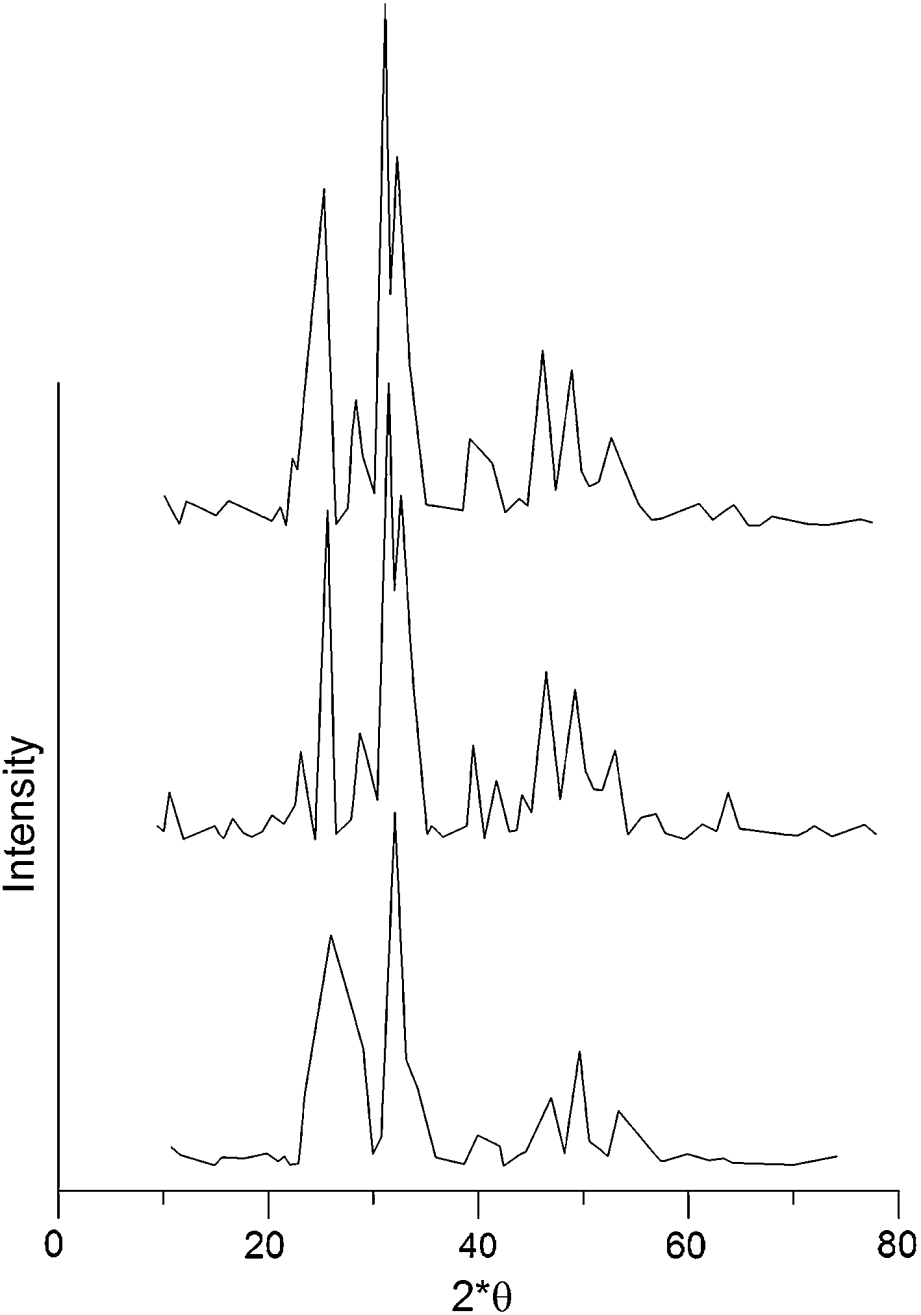
Hydroxyapatite XRD

A crystallographic structure of samples was determined by XRD using the DRON-3 diffractometer using CuK_α_ radiation. The XRD data depicted in Fig. 2 confirms phase purity of the studied samples. On diffractograms of the samples you can observe peaks characteristic for crystallic form of hydroxyapatite, i.e. peaks and their intensities occurring at the angles: 2θ: 25.9–35 %; 31.75–100 %; 32.96–55 %; 39.84–20 %; 46.7–40 %; 49.5–30 %. Except for the peaks characteristic for hydroxyapatite you can observe some peaks confirming the occurrence of calcium carbonate (2θ = 29.4–100 and 39.407–28 %). The carbonate phase probably originated from repeated rinsing the sample with distilled water. A big adsorption affinity of the carbonate ions makes the obtained preparations often contain calcium carbonate [19]. The size of crystallites calculated with the Scherer’s method from half width of the peak for the angle 2Θ = 25.9 was 36 nm for sample 3, 48.4 nm for sample 2 and 28 nm for sample 1. These results agree well with mentioned surface area measurements. Sample 3 has the largest crystallites, since it was prepared under acidic conditions.

**Fig. 3:**
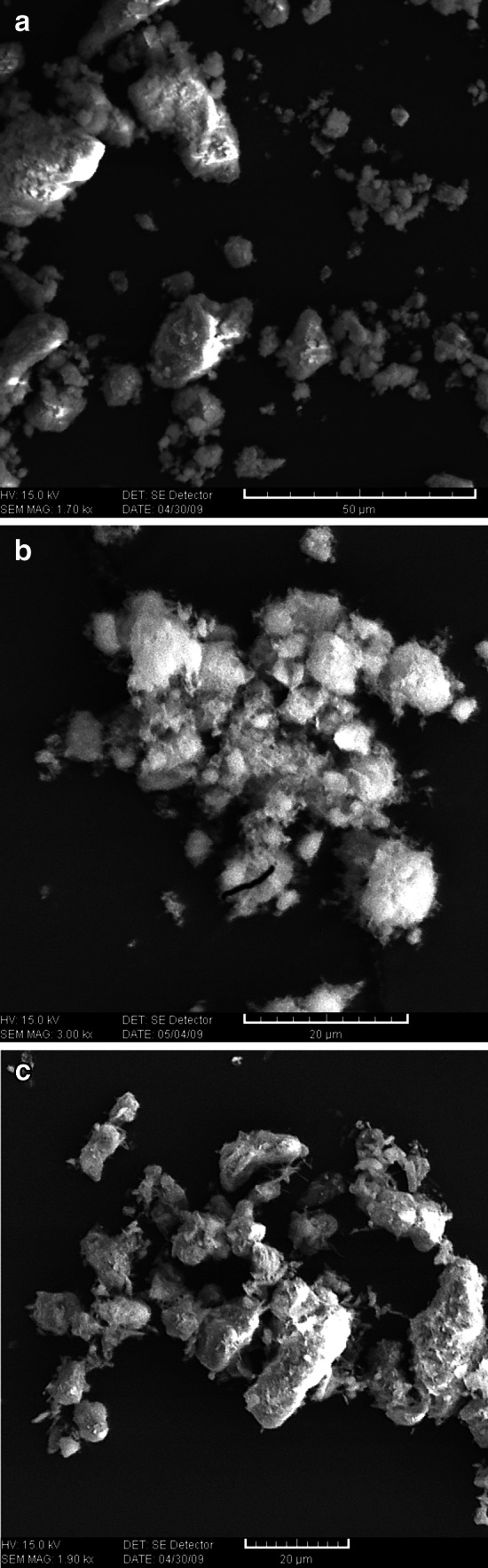
SEM micrograph of hydroxyapatite samples 1 (**a**), 2(**b**), 3(**c**)

On the basis of the photos taken with a scanning microscope (Fig. 3) one can see that in all cases, as a result of the synthesis, we get aggregations of hydroxyapatite molecules, whose sizes reach 20 μm, apart from them smaller molecules of sizes less than 1 μm can be observed. From the sizes of crystallites determined on the basis of half width of XRD file we conclude that hydroxyapatite molecules consist of a huge number of crystals which connect creating a porous structure.

**Fig. 4:**
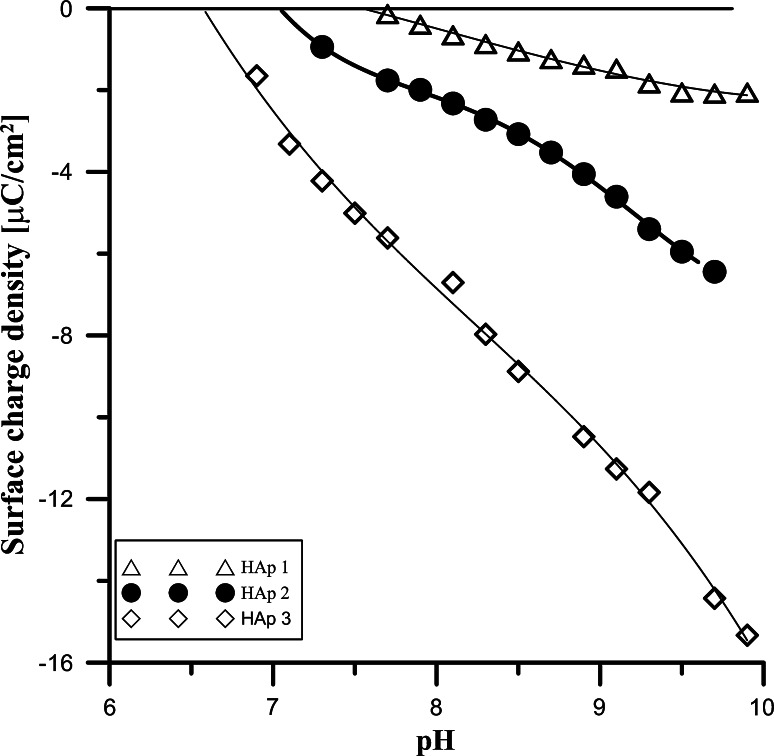
The surface charge density HAP/NaCl solution interface as a function of pH samples 1, 2, 3

The surface charge density as a function of pH for studied samples in 0.001 M NaCl aqueous solution are depicted in Fig. 4 As it can be seen in the studied pH range the values surface charge density are negative, so pHpzc can be determined by extrapolation method. The obtained values for the samples are as follows 7.5 for sample 1, 7.05 for sample 2 and 6.7 for sample 3. Additionally course of the dependence of surface charge density vs. pH shows that acidic properties of surface groups arranges in the following sequence sample 3 > sample 2 sample1. These properties are results of the decreasing amount of calcium ions in the sample determined using EDAX method that is following 29.2 atom % for sample 1, 23.8 at.% for sample 2 and 22.9 at.% for sample 3. The increase of amount of calcium atoms in the sample lead to increase base properties of surface groups that is seen as a shift o pHpzc towards higher pH.

**Fig. 5:**
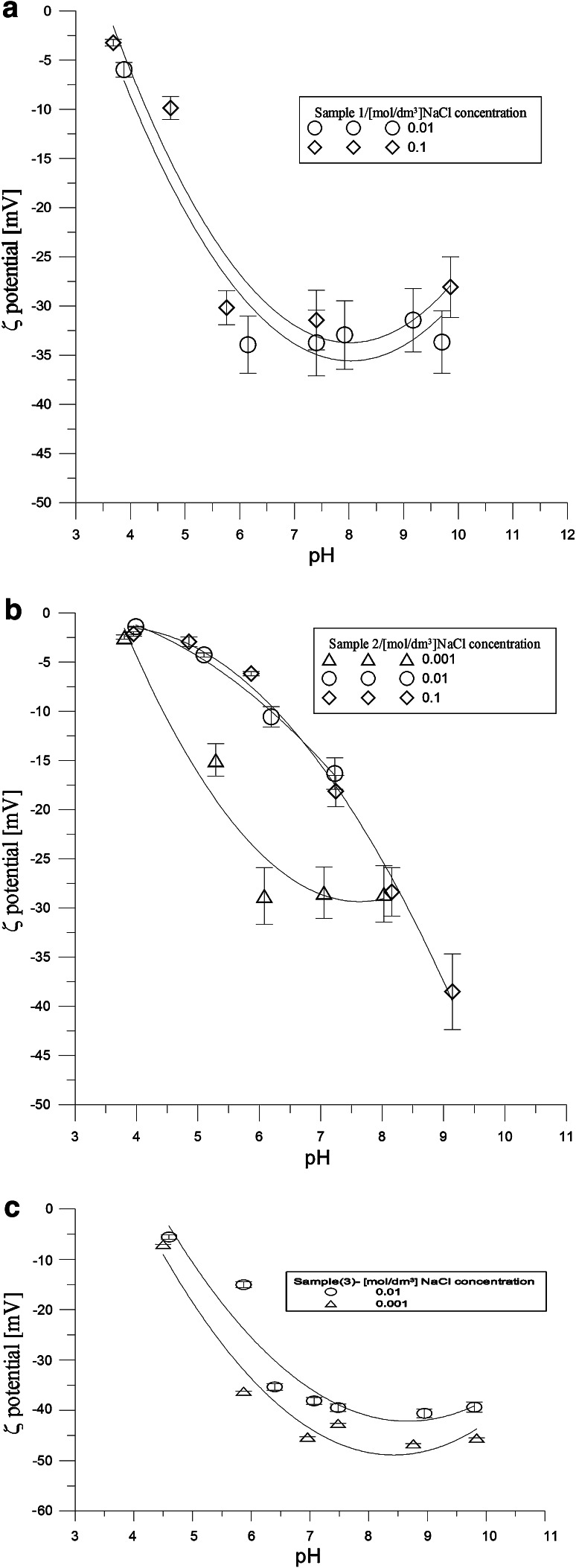
Zeta potential of the hydroxyapatite/NaCl solution interface as a function of pH samples 1 (**a**), 2(**b**), 3(**c**)

The values are consistent with the literature [20].The course of dependence of ζ potential shows that zeta potential decreases with the increase in pH value for all the hydroxyapatite samples (Fig. 5). Extrapolation of the dependence of zeta potential in the pH function lets us assume that pH_IEP_ is about 4.3 for sample 3 so it is over 2 pH units lower than pH_PZC_ for samples 2 and 1 is 3 so it is also lower than pHpzc of the examined samples because the zeta potential is additionally dependent on the part of surface load which is connected with adsorption and desorption of ions of the crystallic lattice: i.e. phosphate and calcium ions. There is a chemical interaction between citric acid and hydroxyapatite, the latter is the structural prototype for the principal inorganic crystalline constituent of teeth and bones.

**Fig. 6:**
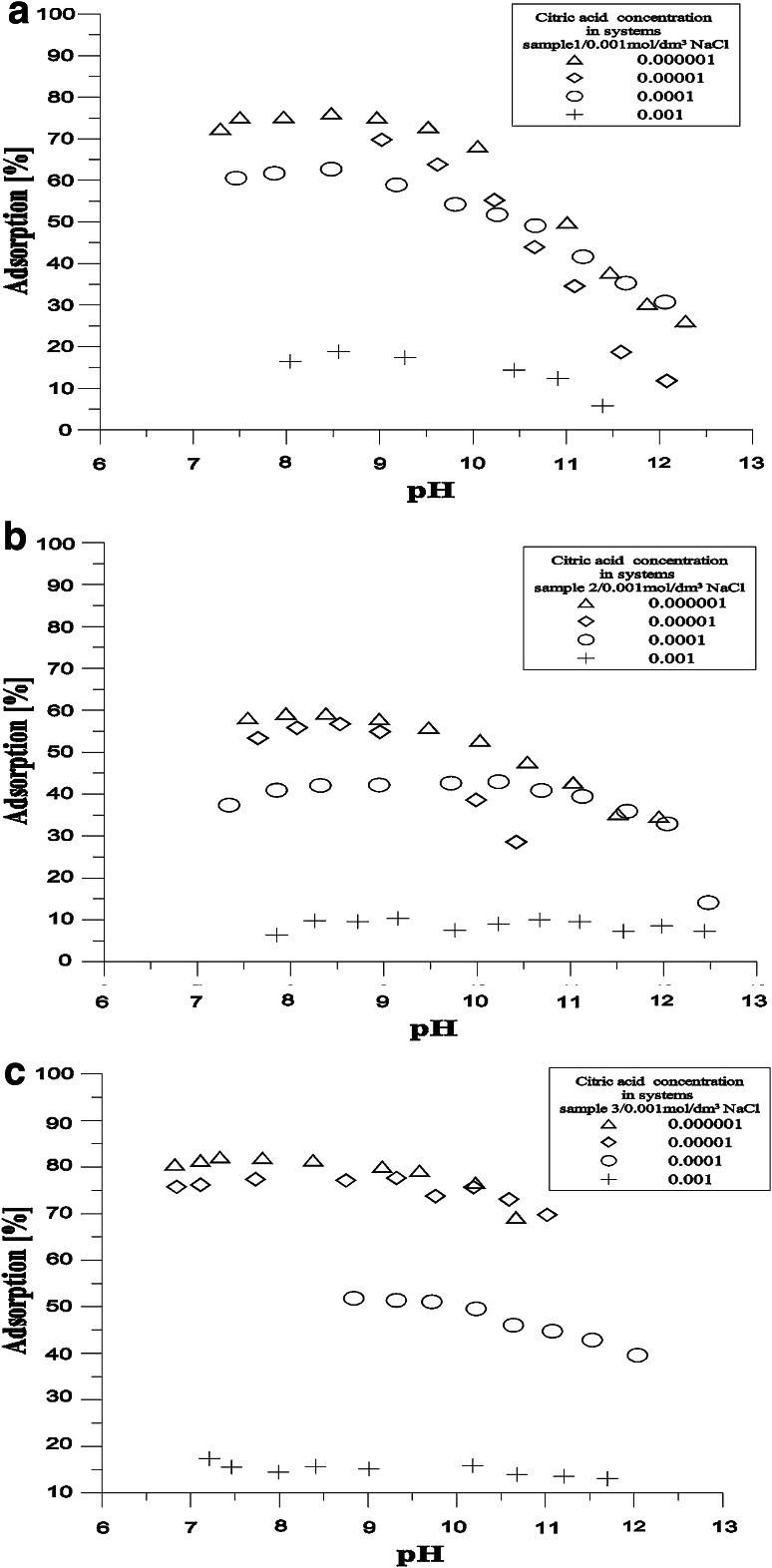
Adsorption of citric acid samples 1 (**a**), 2(**b**), 3(**c**)

Figure 6 presents dependence of citrate ions adsorption on hydroxyapatite/0.001 mol/dm^3^ NaCl solution. As it can be seen the citrate adsorption does not reach 100 % of pH in any of the initial concentrations. In lowest concentrations it reaches only 85 % and that is why it is hard to establish parameters of the adsorption rim such as pH_50 %_ or pH_10–90 %_, because in the examined structure there is no adsorption envelope typical for specific anion adsorption. The proposed mechanism of citric acid adsorption on hydroxyapatite consists in replacing the phosphate ions with citrate ions. Dissociation constants of phosphoric acid pK_a1_ = 2.83, pK_a2_ = 7.2 pK_a3_ = 11.9 and citric acid pK_a1_ = 3.13, pK_a2_ = 4.76 pK_a3_ = 6.40, except for the first step are lower than the respective dissociation constants of citric acid. Therefore replacing the phosphate groups on the surface with the citrate groups will lead to the increase of the density of negatively charged groups on the hydroxyapatite surface. The mechanism of exchanging ions must be excluded because it should lead to a significant increase of negative charge as a result of the increase of citrate adsorption. The citrate ions definitely change the character of the surface of hydroxyapatite-they change its acidic character. Probably it is caused by the creation of the intraspherical complexes as a result of citrate adsorption.

**Fig. 7:**
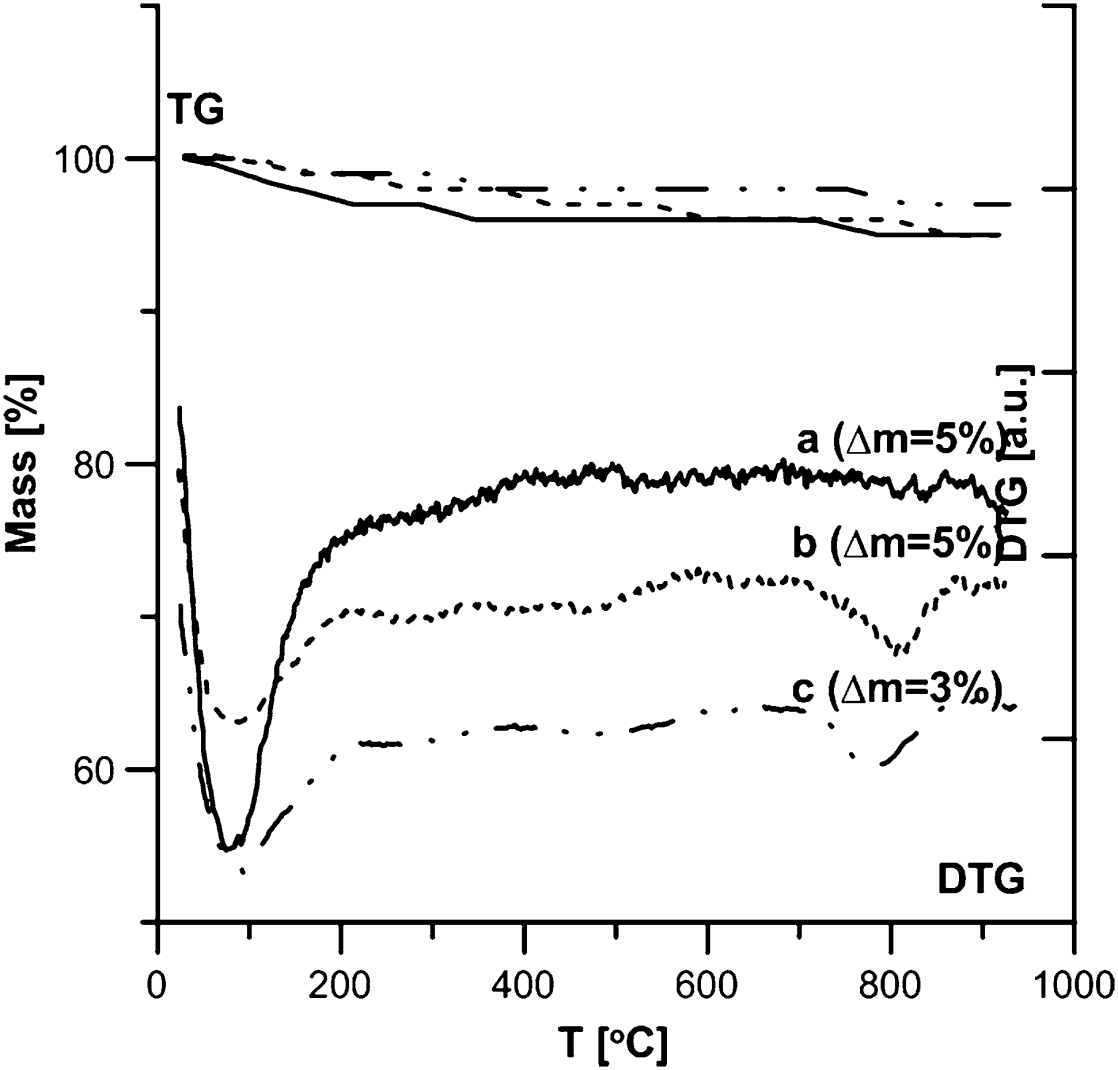
DTA and DTG curves for hydroxyapatite

**Fig. 8:**
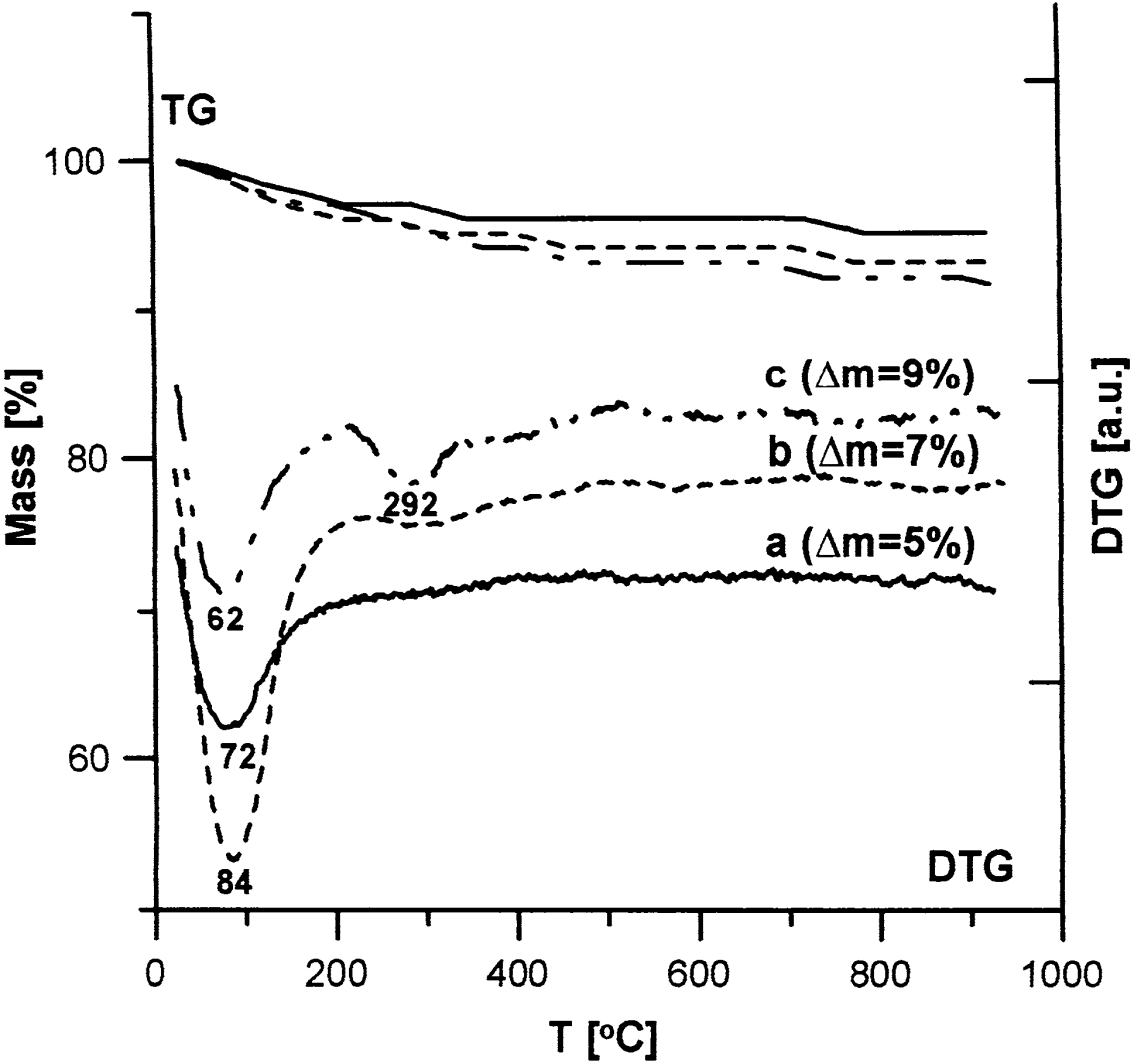
DTA and DTG curves for hydroxyapatite with different citric acid concentrations

**Fig. 9:**
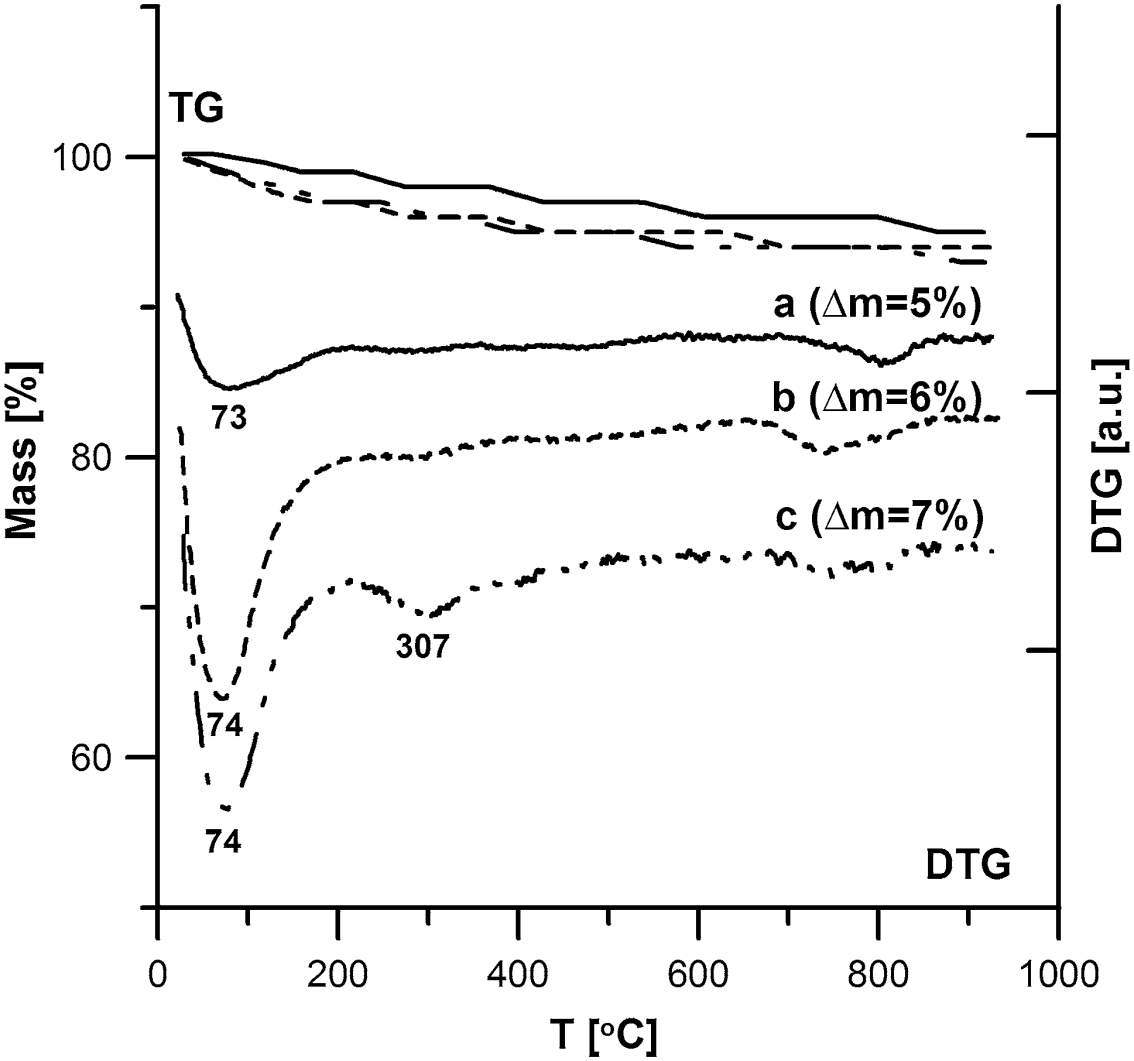
The comparison DTA and DTG curves for hydroxyapatite and hydroxyapatite with citric acid

The structural and adsorptive properties of hydroxyapatite depend on the modification method. Fig. 7 shows the thermogravimetric analysis of the samples synthesized with three methods (samples 1-a, 2-b, 3-c). On the basis of the TG and DTG curves you can conclude that as a result of heating the hydroxyapatite samples within the temperature range 20–950 °C you can distinguish two stages connected with the small weight loss from 3 % for sample 3–5 % for samples 1 and 2. Within the temperature range 20–200 °C the weight loss with the minimum about 72 °C is connected with the endothermal process of removing the hygroscopic and physically adsorbed water. Within 200–950 °C small weight losses are connected with removing carbonates and structural water, chemically combined [21, 22]. The data is compatible with the results obtained by Bianco et al. [18] as well as Tonsueadn et al. [23]. Figures 8 and 9 present the comparison of the TG and DTG curves of the hydroxyapatite samples obtained by method 1 (Fig. 8) and method 2 (Fig. 9) and their samples modified with citric acid solution. Adsorption of citric acid on the hydroxyapatite surface changes not only the active centres on the surface and their energy but it also influences their thermal stability. These effects depend on the concentration of the modifier. In the case of the samples modified by the citric acid solution with 0.00001 M concentration, the termogravimetric curves “b” are similar to those obtained for the base materials on the curve “a”. The bigger peak on the DTG curve in the first part up to 200 °C in relation to pure hydroxyapatites is a sign of removal of the physically adsorbed water and the adsorbate molecules poorly connected with the surface. Evident changes of the DTG curves were observed in the case of modification with acid of 0.001 M concentration. In this case there can be distinguished 3 stages of thermal dissolution –curve C (Figs. 8 and 9). The first stage is connected with removing water and poorly combined citric acid groups on the surface. In the second stage within the temperature range of 200–400 °C the weight loss on the TG curve is accompanied by an explicit peak on the DTG curve with minimal 292 for hydroxyapatite sample 1,307 for sample 2 is connected with precipitation and oxidation of strongly combined organic groups. In the temperature range 400–950 °C oxidation of organic coal to CO_2_ is observed. These changes are similar to the processes during the dissolution of citric acid complexes with metals [24]. This proves creating surface complexes between the molecules of acid and hydroxyapatite and the complexity of the entire process. The loss of intensity on the DTG curve in the temperature range 20–200 °C and the occurrence of the peak give evidence of the increase of the reaction intensity between the surface of hydroxyapatite and citric acid molecules. During DTG experiments a question arose if due to citric acid adsorption calcium citrate is created in reaction with calcium ions of the hydroxyapatite. Analysis of this problem has been carried out but it has been shown that there are too low citric acid concentrations to find it out but it is impossible to exclude such possibility.

## Conclusions

Hydroxyapatite was synthesized by three methods. The influence of synthesis methods on parameters of electrical double layer at the hydroxyapatite/NaCl interface has been investigated. The results of XRD analysis and specific surface area obtained by AFM, SEM and BET indicate the relationship between the synthesis and edl parameters.

1. Investigations of the hydroxyapatite/electrolyte solution system are limited by dissolution of the minerals and pH range 7–11.
2. The synthesis method influences the parameters of electrical double layer at hydroxyapatite/NaCl.
3. The points pH_pzc_ and pH_IEP_ for sample 1 are pH_pzc_ 7.5 and pH_IEP_ 3; for sample 2 pH_pzc_ 7.05 and pH_IEP_ 3, for sample 3 pH_pzc_ 6.7 and pH_IEP_ 3.
4. Derivatographic analysis has shown small influence of temperature on pure substance as well as with citric acid adsorbed, but that the thermal analysis can be used for characterization of structural hydroxyapatite.
5. The adsorption of citric acid occurs as a result of the replacement of the phosphate groups on the surface of hydroxyapatite with the citric ones together with the formation of the intraspherical complexes.
